# Preconception Care Program for Women with Inflammatory Bowel Disease Using Intervention Mapping: A Protocol for Program Development

**DOI:** 10.3390/ijerph17249365

**Published:** 2020-12-14

**Authors:** Young Jin Lee, Yeon Hee Kim, Hae Won Kim

**Affiliations:** 1 Department of Nursing, College of Nursing, Seoul National University, Seoul 03080, Korea; hygiene2@snu.ac.kr; 2 Department of Clinical Nursing, University of Ulsan, Seoul 05505, Korea; kimyhee@amc.seoul.kr; 3 Department of Nursing, The Research Institute of Nursing Science, Center for Human-Caring Nurse Leaders for the Future by Brain Korea 21 (BK 21) Four Project, College of Nursing, Seoul National University, Seoul 03080, Korea

**Keywords:** inflammatory bowel disease, preconception, program development, intervention mapping

## Abstract

The prevalence of inflammatory bowel disease in Korea is rapidly increasing. Women with inflammatory bowel disease have a higher risk of adverse birth outcomes than healthy women, and the magnitude of this risk is related to the severity of the disease at the time of pregnancy. For a woman with inflammatory bowel disease to have a healthy pregnancy, interventions are needed to manage the disease before pregnancy—implying a need for pregnancy planning. In this study, the intervention mapping protocol was used to develop a program for this purpose. This protocol contains the following stages: needs assessment, setting of program outcomes and performance objectives, selection of methods and strategies based on theory, and development of the program and its materials. Through individual in-depth interviews and a literature review, individual and environmental determinants were assessed and six change objectives of the program were set. The methods and practical strategies were developed based on the information-motivation-behavioral skills model, self-efficacy theory, and social support theory. The final program, consisting of four sessions and the corresponding materials, was completed by making revisions based on a content validity assessment by experts and a pilot test. Follow-up studies on the implementation of this program will be conducted in the future.

## 1. Introduction

Inflammatory bowel disease (IBD) is a chronic disease of the gastrointestinal tract that encompasses Crohn’s disease (CD) and ulcerative colitis (UC) [1]. Since IBD is prevalent in younger individuals, from their teens to 30s, the impact of IBD on pregnancy and childbirth deserves attention [2,3,4,5]. IBD was previously a common disease in the West, but its prevalence has increased in East Asian countries in recent years [6]. For instance, in South Korea (hereafter, Korea) the mean annual incidence rate of UC and CD was 0.34 and 0.05 per 100,000 persons, respectively, from 1986 to 1990 [7], but it then increased dramatically to 5.0 and 2.8, respectively, from 2011 to 2014. The incidence of IBD in recent years in Korea is the highest among East Asian countries [6,8].

A meta-analysis of 30 years of data on the birth outcomes of women with IBD showed that they had a 1.85 times higher risk of preterm birth, a 1.36 times higher risk of low birth weight, a 1.57 times higher risk of stillbirth, and a 1.29 times higher risk of birth defects than healthy women [3]. A recent study of the birth outcomes of women with IBD in Korea found results similar to those of international studies; specifically, women with IBD had higher rates of cesarean section, stillbirth, intrauterine growth retardation, and preterm birth [4]. The risk of adverse outcomes in women with IBD is related to disease activity at the time of pregnancy [4,9,10]. If women with IBD are in remission before and during pregnancy, their birth outcomes are similar to those of healthy women. For this reason, interventions to promote disease management before pregnancy should be provided to promote healthy pregnancy and childbirth in women with IBD [2,4,9].

Preconception care refers to the assessment and modification of risk factors affecting women’s health and pregnancy outcomes before pregnancy to improve maternal and child health [11]. In particular, women with chronic diseases require preconception counseling with a healthcare provider to avoid adverse consequences in pregnancy due to the disease itself or pregnancy-induced exacerbation of the disease [12]. Women with IBD have a low level of IBD-related pregnancy knowledge and experience high levels of anxiety about pregnancy as a result of misconceptions about pregnancy [13]. Therefore, the purpose of preconception care for women with IBD is to reduce anxiety about pregnancy and maintain remission for at least 3 months before pregnancy, as well as to provide education about contraception to avoid pregnancy during active periods and unwanted pregnancy [14,15]. Self-management of the disease is necessary to ensure that women with IBD maintain disease control before pregnancy. Social support networks, including family or community members, play an important role in assisting with the health behavior of people with chronic diseases. Social support can alleviate negative factors such as stress and depression and improve health through the exchange of information about the disease, its management, and patients’ emotions [16]. Thus, healthcare providers should assess and strengthen their patients’ social support network. According to a previous study, 61% of patients with IBD in Korea reported difficulty making friends or maintaining relationships [17], and their low level of social support behavior means that it is necessary to establish a support system for interpersonal relationships, including peers, family members, and friends [18].

Although some interventions have been implemented to improve pregnancy-related knowledge in women with IBD [19,20], preconception care interventions by nurses are lacking. Developing and providing preconception care programs that take into account the individual and interpersonal factors of women with IBD will improve the effectiveness of these interventions.

Intervention mapping (IM) is a protocol for systematically developing and implementing theory- and evidence-based health promotion programs to promote behavioral change [21]. IM emphasizes the importance of a social ecological approach to behavioral change, and is useful for identifying individual and environmental determinants and selecting appropriate intervention methods and strategies to address the identified determinants [16,21]. IM has been deployed to promote specific health behaviors such as physical activity enhancement, checkups for cancer prevention, diet management, and smoking cessation [22,23,24,25], and its effectiveness has also been demonstrated for comprehensive health behavior intervention programs such as chronic disease self-management programs [26,27], and reproductive health management [28,29]. In light of the increasing need for preconception healthcare for women with IBD in Korea [4], we planned to develop a preconception care program for women with IBD in Korea using the IM protocol. This is the first study aiming to improve the reproductive health of women with IBD in Korea. By developing a preconception care program, this study is expected to provide the basis for a reproductive health program for women with IBD that can be applied in various clinical settings in the future.

The purpose of this study is to describe the process of systematically developing a preconception care program for women with IBD in Korea using the IM protocol.

## 2. Materials and Methods

IM consists of the following six steps: (1) a logic model of the problem, involving needs assessment; (2) program outcomes and objectives and a logic model of change, which involves setting performance objectives and constructing a matrix of change objectives; (3) program design to choose the theory- and evidence-based intervention methods and practical strategies; (4) program production (focusing on the program’s organization and materials); (5) program implementation planning; and (6) program evaluation planning [21,30]. This paper will describe the first to fourth steps in the IM protocol. 

### 2.1. IM Step 1: Logic Model of the Problem (Needs Assessment)

A planning group was established, which consisted of four persons: a maternity nursing professor, a clinical nursing professor, an IBD nurse specialist, and a doctoral student with experience in IBD patient care and women’s health education and research. The first author (Y.J.L.) met with group members individually or in groups once a week on average to receive advice and to discuss the program development process. Meetings with the planning group contributed to continuing revisions and completion of program development and the evaluation plan based on the needs assessment. In this study, in-depth individual interviews with women with IBD were conducted to confirm the requirements of the program and previous studies were reviewed.

#### 2.1.1. In-Depth Individual Interviews

In order to explore the pregnancy and childbirth experiences of women with IBD in Korea, in-depth individual interviews were conducted with 10 women with IBD who had experienced pregnancy and childbirth. Participating subjects were recruited voluntarily through the IBD Patient Association. This study was approved by the Seoul National University Institutional Review Board (IRB No. 1908/001-007). Participants received an explanation of the purpose of the study and were assured of anonymity and confidentiality. Participants indicated that they understood the purpose and method of the interview and provided written informed consent. The interviews were conducted directly by the researcher (YJL) from August to September 2019. Interviews were conducted at the participants’ homes or in a quiet cafe, and the average duration of the interviews was about 70 min. The interview questions were “What do you think women with IBD need to prepare for pregnancy?” and “What would you have needed in terms of preconception educational content as a woman with IBD?” After each interview, the researcher summarized the interview results and received confirmation from the participant that the summary was correct. All interviews were recorded and transcribed verbatim immediately after the interview. The analysis was performed according to the deductive content analysis process of Elo and Kyngäs [31]. While repeating the transcription line by line, we coded meaningful words, phrases, and sentences related to preconception care, and classified similar codes to derive subcategories. Similar subcategories were grouped and abstracted to derive themes. The process of integrating and analyzing the original data was repeated to reconfirm the derived themes. Reliability was ensured through review by one expert member with experience in qualitative research related to women’s health and two doctoral students in nursing with experience in several qualitative studies.

#### 2.1.2. Literature Review

To identify the personal and environmental factors affecting changes in behavior relevant for the pre-pregnancy management of women with IBD, previous studies related to pregnancy in women with IBD in Korea and abroad were reviewed. Several databases (e.g., Google Scholar, PubMed, RISS, and KoreaMed) were used for the literature review. For papers published after 2010, we searched for “inflammatory bowel disease” “pregnancy” “pre-pregnancy” “preconception” and “reproductive health” as the key words. Papers relevant for this research topic were selected and reviewed.

### 2.2. IM Step 2: Program Outcomes and Objectives, Logic Model of Change

The second step was to establish program outcomes (goals and objectives) and detailed performance objectives to achieve the program objectives [21,30]. This involved creating a matrix that linked the change objectives with performance objectives and behavioral or environmental determinants. We set the goal of the program based on the perceptions and needs for preconception care of women with IBD identified through the individual interviews and literature review in the first stage. Detailed performance objectives were selected to improve IBD management behavior before pregnancy. Individual and environmental factors were determined on a theoretical basis, and the change objectives for each factor were created as a matrix by linking them with detailed action targets.

### 2.3. IM Step 3: Program Design

For the program design stage, we selected intervention methods and strategies based on theory to achieve behavioral change in participants, and selected practical activities for use in the program.

### 2.4. IM Step 4: Program Production

The program production step involves refining the program through pilot testing after preparing a draft program that integrates the results from steps 1 to 3. This step also includes the development of materials [21,30]. We determined the number of program sessions and the detailed content for each session according to participants’ needs, and produced educational materials for use in each session. Video materials to be used in education and auxiliary materials to be provided to the target audience were also created.

## 3. Results

### 3.1. IM Step 1: Logic Model of the Problem (Needs Assessment)

#### 3.1.1. In-Depth Individual Interviews

The characteristics of the women who participated in the individual interviews for needs assessment are shown in Table 1. The average age of the participants was 40.1 years; seven women had been diagnosed with CD and three women with UC. The average age of the women at childbirth was 35.43 years, and the average gestational age was 37 weeks. An analysis of the interviews revealed the absence of a specialized preconception program for women with IBD. The participants said that they had received disease education about IBD, but the content related to IBD and pregnancy was unsatisfactory because the overall education was very comprehensive and pregnancy-related information accounted for a small portion of it. In addition, they also indicated that it was difficult for them to ask sex-related questions because they received IBD education together with male patients. The women with IBD described their experiences of worrying about infertility and adverse pregnancy outcomes. They even avoided marriage or pregnancy for this reason. Women with IBD hesitated to consult gastroenterologists about pregnancy, and were often unwilling to open up to their partner about their condition. The participants wanted to receive education on how to maintain remission before preparing for pregnancy, the importance of gynecological examinations, IBD drugs and pregnancy, anxiety management regarding pregnancy, and disease management during pregnancy. Most participants expressed the opinion that group education would be a suitable format for participants to share their experiences about disease management with each other. However, the opinion was also expressed that individual education would be preferable better because participants may have different attitudes toward pregnancy and health status, and privacy protection may be required due to the discussion of sensitive personal information during education. All participants hoped that the education would be conducted within three sessions. They wanted to receive a workbook and information on patients who had experienced pregnancy and childbirth to reduce their anxiety about having a baby.

#### 3.1.2. Literature Review

**Figure 1:**
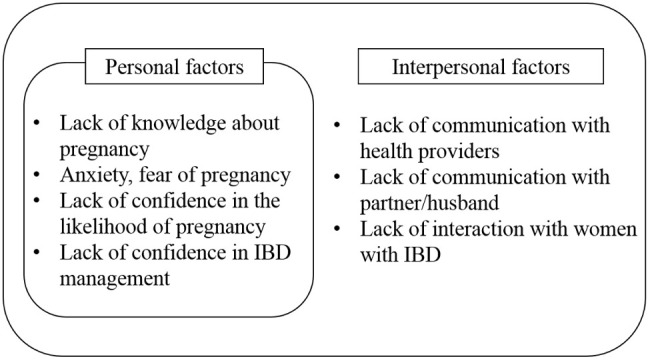
Personal and interpersonal factors related to preconception care of women with inflammatory bowel disease (IBD) based on a needs assessment through interviews and a literature review.

By reviewing several previous studies, we confirmed that most women of childbearing age with IBD have considerable anxiety about pregnancy due to the exacerbation of the disease during pregnancy, potential difficulties in becoming pregnant, and the possible inheritance of IBD [32,33,34]. This anxiety is related to misinformation about pregnancy, and the resultant anxiety contributes to voluntary childlessness [13,35]. Therefore, it was found that the provision of correct information should be prioritized in interventions for IBD women. Figure 1 shows the personal and interpersonal factors related to preconception care of women with IBD based on the needs assessment through interviews and a literature review.

### 3.2. IM Step 2: Program Outcomes and Objectives: Logic Model of Change

The goal of this program was “women with IBD should be prepared before pregnancy” and the program objective was “women with IBD should be prepared for pregnancy while maintaining remission for at least 3 months”. The performance objectives of this program were divided into six areas: (1) practice pregnancy planning; (2) take medicine as prescribed; (3) perform regular exercise; (4) consume healthy foods; (5) stop smoking and drinking alcohol; and (6) receive counseling about pregnancy with a healthcare provider. The determinants were divided into personal and interpersonal areas. Factors influencing behavioral change in each area were derived through the health promotion theory. In this study, based on the needs assessment in step 1, the personal domain was derived by applying the information–motivation–behavioral skills (IMB) model and self-efficacy theory. Knowledge and attitude/motivation were drawn from the IMB model [36], and self-efficacy was adopted as a determinant from self-efficacy theory [37]. As an interpersonal determinant, social interaction (from social support theory) was derived as a related factor [38]. The matrix of this program is shown in Table 2.

### 3.3. IM Step 3: Program Design

The intervention methods, related theories, and practical strategies to improve the knowledge, attitudes, self-efficacy, and social support of women with IBD are presented in Table 3. In order to improve the IBD-related pregnancy knowledge of women with IBD, information provision was selected as a method based on the IMB model [29,39]. For practical applications, it was decided to provide information through small-group lectures, text messages, and educational workbooks. Bandura’s (1977) concepts of efficacy expectations, performance accomplishments, vicarious experience, verbal persuasion, and emotional arousal were applied to improve the attitudes, motivations, and self-efficacy of women regarding preconception care [37]. As strategies to accomplish these goals, individual telephone coaching, presentations on the pregnancy and childbirth experiences of women with IBD through video, small-group discussions, and a preconception care diary were chosen. Finally, to promote social interaction among women participating in the program, we decided to share experiences of management behavior before and after each session. In addition, tasks to encourage counseling and communication about preconception care with partners or health providers were performed.

### 3.4. IM Step 4: Program Production

#### 3.4.1. Development of the Program

##### Preliminary Program Development

A structured preliminary program including the educational content and methods was created based on the participants’ needs and previous research considerations [14,40,41]. The initial program consisted of three sessions, and the topics were “pregnancy outcomes and IBD management for women with IBD”, “prenatal management from pregnancy to delivery”, and “sharing pregnancy experiences and stress management.”

##### Program Validation

Seven experts reviewed the content validity of the preliminary program. Maternal nursing professors, gastroenterologists, an IBD nurse specialist, two certified wound care nurses, and two gastroenterology nurses with more than 10 years of experience evaluated the program’s goals, content and composition, schedule, and educational methods. For each item, content validity was verified based on a score of 3 points or more for the content validity index (CVI), which consisted of a 4-point scale (1 = not valid, 2 = somewhat valid, 3 = valid, 4 = very valid). The percentage of responses was calculated. The CVI value for each question ranged from 0.79 to 0.96. Experts recommended that redundant educational content be deleted and that information on drug use during pregnancy and postpartum care, including breastfeeding, be added. The program was revised to reflect additional expert opinions.

##### Pilot Testing

The revised program was implemented for two women with IBD with no pregnancy experience, and the final revision of the program was made by collecting opinions about the schedule, training content, and training methods from the women. One of the women who participated in the preliminary study was aware of the characteristics of IBD patients because she had experience as an executive of the IBD Patient Association. They expressed that it would be desirable to add information on the current health status of the children of women with IBD. In addition, they wanted to be educated on gynecological diseases, and hoped that activities for future babies would be carried out as small group activities. They said that the weekend would be better than weekdays for the education sessions, considering participants’ work and academic status.

##### Final Program

The program was completed by revising items based on the expert review and pilot test results. The final program consisted of four sessions (80 min each), and each session was organized in the order of introduction, education, and closing. During the introduction and closing phases, participants share their experiences and opinions in small group discussions for approximately 30 min. The theme of the first session is “Managing myself for IBD” during which reproductive health for women with IBD and IBD management methods are introduced. The second session is “Smart preparation for healthy pregnancy?” which focuses on pre-pregnancy preparation for women with IBD and their spouses (partners), including pregnancy planning and contraception to avoid pregnancy during active periods. The theme of the third session is “Smart preparation for a healthy childbirth” which provides education on management until childbirth. The last session, with the theme of “Get ready to have my baby!” includes video interviews of women with IBD who experienced pregnancy and childbirth and messages of encouragement related to pregnancy. Table 4 shows the composition and details of the final program.

#### 3.4.2. Development of Materials

##### Workbook

We developed a booklet entitled “Happy pregnancy and childbirth preparation for women with IBD” to complement the education delivered orally to the women and to enable self-learning of the educational content after the educational sessions. The booklet met the evaluation criteria for content, literacy level, graphic illustrations, lists, tables, charts, layout and typography, learning stimulation and motivation, and cultural appropriateness by referring to 22 elements of the Korean version of the Suitability Assessment of Materials [42]. A maternal nursing professor, gastroenterologist, obstetrician and gynecologist, and IBD nurse specialist reviewed the content of the workbook and made revisions.

##### Preconception Care Diaries

We created a preconception care diary that IBD women can use for 6 months to help them maintain remission for more than 3 months before pregnancy. The contents of the diary include IBD symptoms, sleeping time, mood, taking IBD medications, taking folic acid, exercising, the type of food consumed, and the food that caused symptoms to worsen, to help participants understand their healthcare status on a daily basis [43]. The date of outpatient visits and the administration date of regularly administered injection drugs, such as biologic agents, can be displayed on the monthly calendar. In addition, for reproductive health management, the timing and duration of menstruation and the use of contraception can be displayed. A maternal nursing professor, gastroenterologist, obstetrician, IBD nurse specialist, and women with IBD evaluated the diary. As a result, it was evaluated suitable for preconception care in women with IBD. 

##### Video Clips

We interviewed a woman who succeeded in pregnancy and childbirth after having a stoma, and a woman who experienced worsening symptoms during pregnancy, but gave birth to a healthy child due to appropriate medication, and recorded videos that lasted 10 min each. The content of the videos included “IBD diagnosis and disease fighting process”, “opinions about pregnancy before becoming pregnant”, “healthcare during pregnancy”, “delivery process”, “current health status and health status of my child”, “the meaning of pregnancy and childbirth as a person with IBD”, and “advice from an experienced mother to prospective mothers”. In addition to the interviews, messages of encouragement from six patients were added and edited, and the video production was completed.

## 4. Discussion

This study describes the development of a preconception care program for women with IBD using the IM protocol, with the goal of focusing on behavioral change for chronic disease management beyond traditional knowledge-based education-driven education. IM is useful for changing health behaviors of chronically ill patients, as it enables clinicians to set detailed change objectives, select an intervention method based on theory, and evaluate the effect of the intervention in changing patients’ behavior. Therefore, this study is very meaningful in that it is the first study to attempt to prepare women with IBD in Korea for pregnancy and that it presents the development of a program for behavioral change. Preconception education should be offered to women with chronic disease as soon as the disease is diagnosed to reduce any excessive anxiety about pregnancy and ensure a healthy pregnancy [33]. Any woman of childbearing age is subject to preconception education, and the content is not limited to pregnancy: it includes the overall reproductive health of women with IBD. In addition, to promote self-management of diseases, a diary was distributed as Supplementary Material (File S1). Therefore, the target of this program may include women of childbearing age as well as those preparing for pregnancy in the near future.

Previous studies have investigated the pregnancy and childbirth outcomes and the degree of pregnancy knowledge of Korean women with IBD [4,44], but the educational content and methods required by actual patients were not previously identified. Therefore, a strength of this study is that we met women with IBD in person to provide insights for the development of this program and conducted in-depth interviews to confirm the knowledge and pregnancy-related attitudes of women with IBD in Korea. Although it is ideal to recruit the same proportion of women diagnosed with CD and UC when interviewing, as a result of voluntary participation, most of the participants were women diagnosed with CD. The preconception education for IBD women includes both CD and UC patients, and there is no difference in the knowledge of pregnancy and preconception behaviors according to the diseases [5,35,44]. In the results of this interview, the contents that participants wanted for preconception education are similar to those already recommended in previous studies [35,41]. Therefore, it seems appropriate to prepare educational content based on the results of the interviews. In addition, as a pilot test was conducted for actual women with IBD at the program production stage, additional educational content, and educational methods desired were reflected in the request for revision and were fulfilled. By reflecting the opinions of the subjects who are particularly familiar with patients, it can be observed that the program was developed by knowing the needs of the subjects indirectly and applying those to ensure participation in the program. As such, the target-centered program developed based on a needs assessment is expected both to achieve the goal of education and to increase participants’ satisfaction.

The theories applied in this program ultimately aim to improve health behavior. The IMB model explains that when individuals have sufficient information about health behavior and motivation to change their behavior, they are more likely to make behavioral changes [36,39], and self-efficacy is a necessary element for health behavior [37]. The effectiveness of self-management programs using the IMB model and self-efficacy theory in IBD patients has been proven in prior studies [45,46,47]. Therefore, these theories form an appropriate basis for a program for women with low IBD-related knowledge and high anxiety about pregnancy to help them consistently manage disease and practice preconception care behavior.

Preconception care must be done with one’s partner, so improving women’s relationships and communication with their partners is paramount. However, most women with IBD experience an impaired body image due to the disease and have sexual function problems, which affect their relationships with their partners [48]. In particular, previous studies have found that IBD patients have difficulties in sexual communication with their partners [49], making it necessary to implement interventions to assess and improve the degree of women’s sexual communication with their partners. Thus, this program aimed to improve social interaction. Previous studies have shown that women with IBD are passive in counseling related to sex, pregnancy, and contraception with medical staff [49,50]. No report has yet investigated the preconception counseling status of women with IBD in Korea, but the results of the needs assessment in the first step of this process revealed that most of them did not consult with a gastroenterologist before pregnancy. For this reason, it was necessary to help women with IBD actively receive counseling with a gastroenterologist to undergo tests such as a colonoscopy and to consult about taking drugs to prepare for pregnancy. When the program is implemented in the future, with education on the importance of counseling for pregnancy and encouragement of women to receive counseling before pregnancy, it is expected that communication with medical staff will be strengthened, both in pregnancy and for disease management in the future.

The program included both small group training and individual interventions. In the process of sharing experiences and encouraging participants with chronic diseases, it is expected that vicarious experiences and emotional support will play a positive role in enhancing self-efficacy [46]. Since the symptoms of IBD and the attitudes and knowledge about pregnancy may vary from person to person, we intend to conduct individual phone coaching. We will assess individual IBD symptoms and management, pregnancy awareness and health behavior, and disease management barriers, and provide customized information and emotional support accordingly. By practicing communication and individual phone coaching with providers, this program is expected to help in improving communication with health providers in the future. It is necessary to consider various methods of providing education to encourage the participation of the patients. In particular, because most of the patients with IBD are young, it may be difficult for them to participate directly, due to work or studies. Audio-visual materials, such as PowerPoint and video, were produced as the material of this program, meaning it can be provided face-to-face or online. As the effectiveness of online preconception education has been proven [20], it is necessary to consider the online form when planning on how to provide the program.

The material developed for this educational program not only provides information, but also aims to enhance participants’ attitude, motivation, and self-efficacy. The video that introduces a patient’s pregnancy experiences will reduce anxiety about pregnancy and foster confidence in pregnancy preparation, as it presents a patient and her child who experienced a healthy pregnancy and childbirth. The preconception diary will contribute to daily achievements in disease management by encouraging participants to write about their self-management status. In addition, since the items in this diary were developed based on previous research about self-management of IBD [51,52,53,54,55], writing a diary is considered an opportunity for participants to learn how to manage disease on their own. For IBD patients, keeping a diary can improve self-efficacy in disease management and promote symptom relief [51]. Therefore, this diary is expected to be an important tool to help the participants’ disease management. 

It is very meaningful that various experts participated in the program development process. The necessity of a multidisciplinary approach to pregnancy-related interventions for women with IBD has been emphasized [40]. Nurses mainly caring for IBD patients in the ward, an IBD nurse specialist, gastroenterologists, obstetricians, nurses who provide wound and ostomy care, and nursing professors participated in verifying the validity of the content of this program and in reviewing the educational materials. The accuracy of the program’s content can be verified with the participation of a large number of experts who know the latest information on how to educate women with IBD. Nurses are health professionals with an understanding of patients and expertise in IBD, and are powerful resources for providing patients with the information they want and improving their health management capabilities [46]. Therefore, this program is a model of nursing education developed and led by nurses for preconception care of women with IBD, thereby promoting the expansion of nurse-run education programs in the future.

However, despite the many strengths of this program, it also has some limitations. The individual interviews mainly focused on women with IBD, and information on their environment (including the family) were indirectly confirmed. Since women are not the only people involved in pregnancy, it is important for both women and men to manage their health before pregnancy [11]. Including men in pre-pregnancy management programs can reduce the risk of high-risk pregnancy; therefore, it is necessary to develop a program that also targets the husbands or partners of women with IBD [56]. Therefore, in a further study, we recommend meeting directly with family members, partners, and medical staff to reflect the need for comprehensive and inclusive education. 

## 5. Conclusions

This study was conducted to help Korean women with IBD, the prevalence of which is increasing in Asia, prepare for pregnancy, while maintaining remission. Based on the educational content and methods required by women with IBD, the program development process, according to steps 1–4 of the IM protocol, was described. In particular, it is very meaningful that the program was developed based on a rigorous theoretical background. In the future, we plan to implement the intervention and to evaluate its effectiveness. If the effectiveness of the program is verified for Korean women with IBD, we expect that it will be extended and applied to women with IBD from other cultures in the future.

## Figures and Tables

**Table 1:** Characteristics of the participants in in-depth interviews.

No	Age	Diagnosis	Age at Last Childbirth	Number of Children	Gestation (Weeks)	Childbirth Method	Interview Time (min)
1	37	CD	35	2	37, 34	NSVD	67
2	39	CD	32	1	39	C/sec	75
3	40	CD	37	1	37	C/sec	60
4	39	CD	35	2	33 *	NSVD	56
5	39	CD	35	1	37	NSVD	74
6	39	CD	37	1	39	C/sec	90
7	41	UC	40	2	38, 37	NSVD	60
8	45	CD	36	2	39, 35	NSVD	90
9	41	UC	39	1	38	C/sec	50
10	41	UC	35	2	41, 38	C/sec	80

Note: CD = Crohn’s disease, UC = ulcerative colitis, NSVD = normal spontaneous vaginal delivery, C/sec = cesarean section, *: twin.

**Table 2:** Matrix of the preconception care program for women with IBD.

Program Goal: Women with IBD Should be Prepared before Pregnancy				
Program Objective: Women with IBD Should Be Prepared for Pregnancy While Maintaining Remission for at Least 3 Months.				
Performance Objectives	Personal Determinants			Interpersonal Determinant
	Knowledge (K)	Attitude/Motivation (AM)	Self-Efficacy (SE)	Social Interaction (SI)
PO1. Practice pregnancy planning	K. 1a. Understand and explain fertility and pregnancy outcomes of women with IBD. K. 1b. Understand and explain the importance of planned pregnancy. K. 1c. Understand and explain types of contraception.	AM. 1a. Express positive feelings about pregnancy with IBD. AM. 1b. State an increased motivation for planning pregnancy. AM. 1c. State an increased motivation for contraception to avoid unwanted pregnancy.	SE. 1a. Express confidence in staying healthy for pregnancy. SE. 1b. Express confidence in the ability to practice pregnancy planning. SE. 1c. Express confidence in the ability to practice contraception to avoid unwanted pregnancy.	SI. 1a. Communicate with husband/partner about the appropriate time for pregnancy SI. 1b. Communicate with husband/partner about preconception behavior. SI. 1c. Communicate with husband/partner about the contraceptive method to avoid unwanted pregnancy.
PO2. Take medicine as prescribed	K. 2. Understand and explain what types of IBD medications can be taken before pregnancy.	AM. 2a. Express positive feelings about taking IBD medicine before pregnancy. AM. 2b. State an increased motivation to take medicine as prescribed.	SE. 2. Express confidence in taking medicine as prescribed.	SI. 2a. Communicate with peers about their experience of medication. SI. 2b. Communicate with a healthcare provider about medication.
PO3. Perform regular exercise	K. 3a. Understand and explain the effects of exercise for symptom relief of IBD. K. 3b. Understand and explain whether exercise is important when preparing for pregnancy.	AM. 3a. Express positive feelings about regular exercise. AM. 3b. State an increased motivation to exercise regularly.	SE. 3. Express confidence in exercising regularly.	SI. 3. Communicate with peers about experiences of exercising.
PO4. Consume healthy foods	K. 4a. Understand and explain what foods make IBD symptoms worse. K. 4b. Understand and explain the importance and dosage of folic acid to take appropriately depending on the IBD condition.	AM. 4a. State an increased motivation to avoid foods that make IBD symptoms worse. AM. 4b. State an increased motivation to take folic acid.	SE. 4a. Express confidence in avoiding foods that make IBD symptoms worse. SE. 4c. Express confidence in taking folic acid.	SI. 4a. Communicate with peers about experiences of dietary management for IBD. SI 4b. Communicate with a healthcare provider about the appropriate folic acid dosage for pregnancy.
PO5. Stop smoking and drinking alcohol	K. 5. Understand and explain the need to stop drinking alcohol and quit smoking before pregnancy.	AM. 5. State an increased motivation to stop drinking alcohol and quit smoking.	SE. 5. Express confidence in stopping alcohol and quitting smoking.	SI. 5. Communicate with the peers about experiences of stopping drinking alcohol and quitting smoking.
PO6. Receivecounseling aboutpregnancy with a healthcare provider.	K. 6. Understand and explain the importance of counseling with a healthcare provider before pregnancy for women with chronic conditions.	AM. 6a. Express positive feelings about counseling about pregnancy with a healthcare AM. 6b. State an increased motivation for gastroenterological and gynecological examinations before pregnancy.	SE. 6a. Express confidence in communicating with a healthcare provider SE. 6b. Express confidence in gastroenterological and gynecological examinations	SI.6. Communicate with a healthcare provider about the appropriate time for pregnancy.

**Table 3:** Theoretical methods, strategies, and practical applications.

Theoretical Basis for Determinant	Determinant	Change Objective	Methods (Theory)	Strategies and Practical Applications
Information–motivation–behavioral skills (IMB) model	Knowledge	Increased knowledge of pregnancy and preconception care for women with IBD	∙ Providing information (IMB model)	∙ Providing information: small group lectures, social networking service messages, workbook
	Attitude/motivation	Positive attitude toward pregnancy and pre-pregnancy preparation, and promotion of motivation to prepare for pregnancy	∙ Emotional arousal (self-efficacy theory) ∙ Verbal persuasion (self-efficacy theory) ∙ Vicarious experience (self-efficacy theory)	∙ Writing a letter to their child to be born: workbook ∙ Supporting and motivating: individual tele-coaching ∙ Sharing the experience of women with IBD who have experienced pregnancy and childbirth: video clip
Self-efficacy theory	Self-efficacy	Confidence in preconception management and improvement in IBD disease management in women with IBD	∙ Performance accomplishments (self-efficacy theory) ∙ Vicarious experience (self-efficacy theory) ∙ Verbal persuasion (self-efficacy theory)	∙ Participants keep records and monitor preconception behavior : Preconception diary. ∙ Sharing positive and negative experiences of others: small group discussion ∙ Identifying difficulties of disease management in everyday life and finding solutions: individual tele-coaching
Social support theory	Social interaction	Improvement in communication skills with peers, husband/partner, and health providers	∙ Enhancing communication (social support theory)	∙ Support from peers during small group discussions ∙ Talking to husband/partner or health provider about preconception care: home activity

**Table 4:** Content of the preconception care program for women with IBD.

Session	Theme	Methods	Topics and Activities	Tools/Materials
1	Managing myself for IBD	Lecture	- IBD and current treatment trends - Reproductive health in women with IBD - Fertility of women with IBD - Birth outcomes of women with IBD - 10 commandments of IBD management in daily life - Guideline for reproductive health diary	·PowerPoint ·Workbook
		Group work	- Sharing disease management experiences - Checking disease-related knowledge	·Workbook
2	Smart preparation for healthy pregnancy	Lecture	- Preconception behavior of women with IBD - Medication before pregnancy - Laboratory and imaging exams before pregnancy - Importance of pregnancy planning, contraception - Men’s preconception care	·PowerPoint ·Workbook ·Video clip
		Group work	- Sharing experiences of symptom management - Sharing impressions	·Workbook ·Preconceptiondiary
		Tele-coaching	- Identifying difficulties of disease management in everyday life and finding solutions	·Coaching guide ·Preconception diary
3	Smart preparation for healthychildbirth	Lecture	- Management during pregnancy - Laboratory and imaging exams during pregnancy - Childbirth methods for women with IBD - Postpartum care - Breastfeeding and medication - Immunization of the newborn	·PowerPoint ·Workbook
		Group work	- Talking about reproductive health management and current pre-pregnancy health behavior practiced by participants - Sharing thoughts about care during pregnancy - Checking delivery knowledge related to IBD - Sharing impressions	·Workbook ·Preconceptiondiary
4	Get ready to have my baby!	Lecture	- Sharing the experience of women with IBD who have experienced pregnancy and childbirth	·PowerPoint ·Workbook ·Video clip
		Group work	- Talking to each other about changes in thoughts about pregnancy - Writing a letter to my child to be born - Sharing own feelings about the whole program	·Workbook ·Preconception diary
		Tele-coaching	- Identifying difficulties of disease management in everyday life and finding solutions	·Coaching guide ·Preconception diary

## Supplementary Materials

The following are available online at https://www.mdpi.com/1660-4601/17/24/9365/s1. File S1: Happy pregnancy and childbirth preparation for women with IBD.
