# Supplementary material for: Preconception Care Program for Women with Inflammatory Bowel Disease Using Intervention Mapping: A Protocol for Program Development
Source: Int J Environ Res Public Health. 2020 Dec 14;17(24):9365. doi: 10.3390/ijerph17249365 (PMC7765051; doi:10.3390/ijerph17249365)
Supplement: Supplementary file 1 [file ijerph-17-09365-s001.pdf]

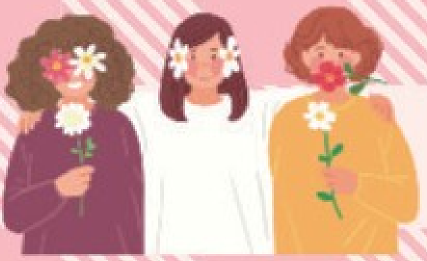

Happy pregnancy and childbirth preparation for women with IBD

My Incredible Baby & pregnancy care Diary

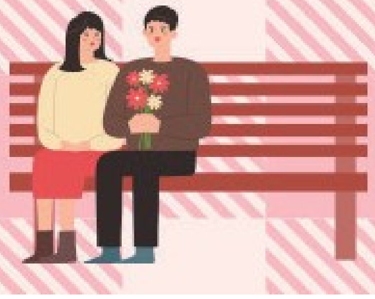
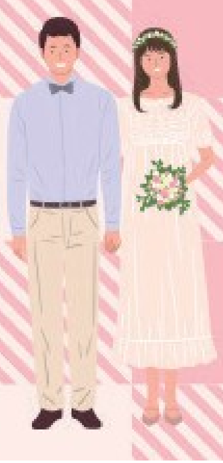
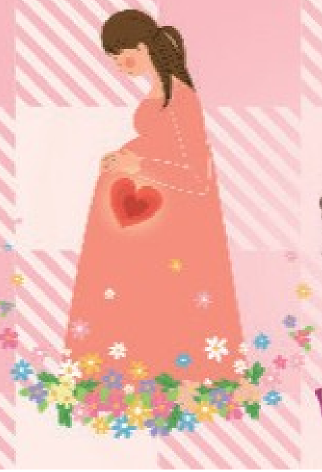
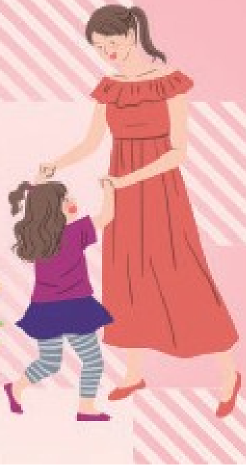

Contents

ONE. Managing myself for IBD

1. Reproductive health in women with IBD

2. Fertility and birth outcomes of women with IBD

3. 10 commandments of IBD management in daily life

TWO. Smart preparation for healthy pregnancy

1. Importance of pregnancy planning

2. Preconception behavior of women with IBD

3. Partner's preconception care

4. Contraception

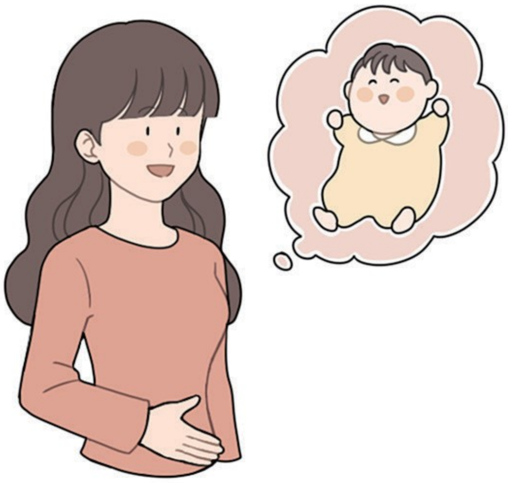

THREE. Smart preparation for healthy childbirth

1. IBD management during pregnancy
2. Laboratory and imaging exam during pregnancy
3. Daily life management during pregnancy
4. Childbirth methods for women with IBD
5. Breastfeeding and medication
6. Postpartum care
7. Immunization of the newborn

FOUR. Get ready to have my baby!

1. Summary of this program
2. Sharing the experience of women with IBD who have experienced pregnancy and childbirth
3. ‘To my child to be born’

ONE. Managing myself for IBD

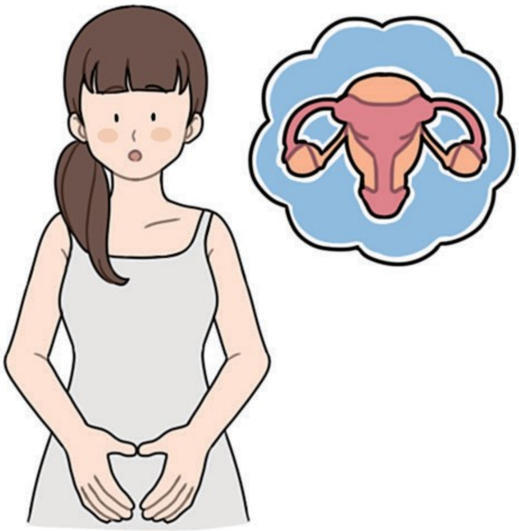

In this chapter, we will look at the reproductive health problems that women with inflammatory bowel disease often experience.

In addition, we will introduce infertility, inheritance, pregnancy complications, and the relationship between pregnancy and disease in relation to pregnancy in women with inflammatory bowel disease.

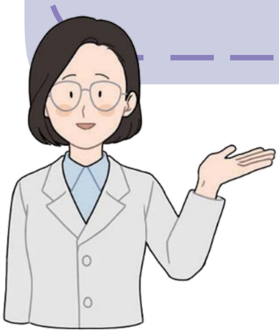

TWO. Smart preparation for healthy pregnancy

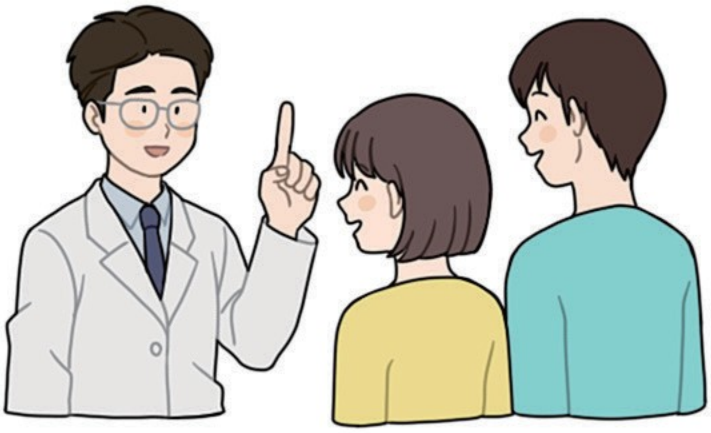

To become pregnant, you need to be prepared at least 3 months in advance. In this chapter, we will introduce you to specific methods of preparing for pregnancy. We will introduce the importance of planned pregnancy and find out how you and your spouse (partner) should prepare specifically. Finally, here are some contraception methods for avoiding unplanned pregnancy.

Remember, healthy babies are born from healthy mothers!

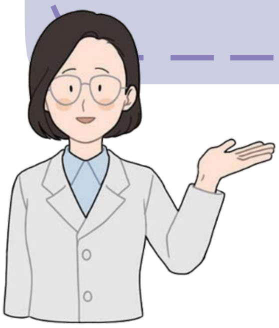

THREE. Smart preparation for healthy childbirth

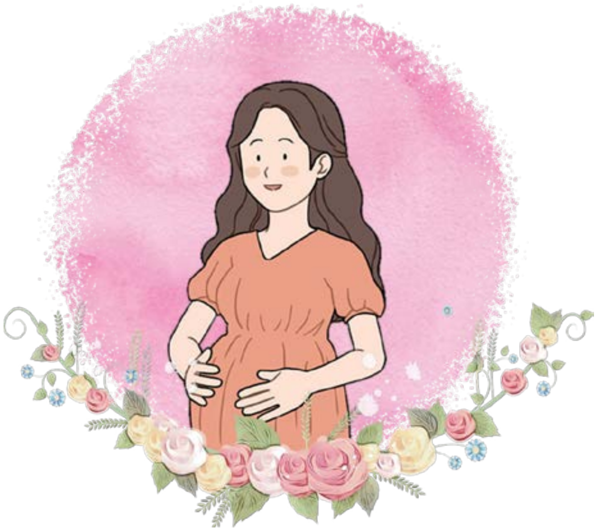

In order to maintain a healthy pregnancy and give birth, you must continue managing disease during pregnancy and after delivery.

In this chapter, we will introduce not only medications and tests for the management of inflammatory bowel disease during pregnancy, but also prenatal tests that are received during pregnancy, delivery methods and breastfeeding.

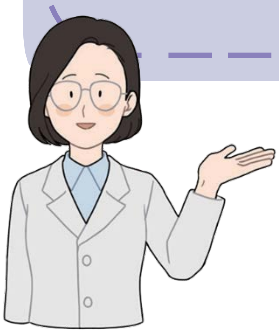

FOUR. Get ready to have my baby!

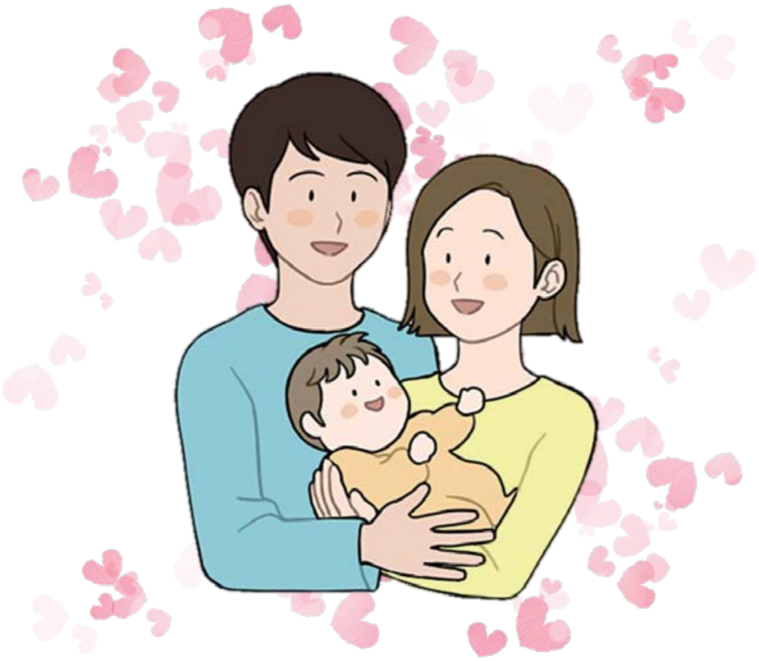

This time, we will summarize the contents of the program for women with inflammatory bowel disease, and listen to the experiences of patients who have been diagnosed with inflammatory bowel disease and have been pregnant and given birth.

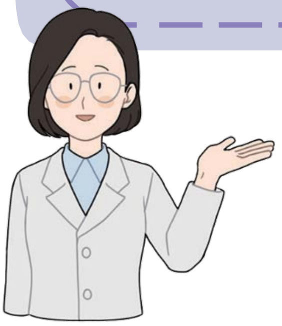

My Incredible Baby & Pregnancy Care Diary

Date. . . .

Am Pm : ~ Am Pm :

IBD medication Folic acid

Exercise time ( ) mins

Normal form loose stool diarrhea Total No ( )

Mood state 1 —2 —3 —4 —5 happy

	Food type	Bowel symptoms
Breakfast		
Lunch		
Dinner		
Snack		
